# Pre-graduate and post-graduate education in personalized medicine in the Czech Republic: statistics, analysis and recommendations

**DOI:** 10.1186/1878-5085-5-22

**Published:** 2014-12-15

**Authors:** Jiri Polivka, Jiri Polivka, Marie Karlikova, Ondrej Topolcan

**Affiliations:** Department of Histology and Embryology, Faculty of Medicine in Pilsen, Charles University in Prague, Husova 3, Pilsen, 301 66 Czech Republic; Biomedical Centre, Faculty of Medicine in Pilsen, Charles University in Prague, Husova 3, Pilsen, 301 66 Czech Republic; Department of Neurology, Faculty of Medicine in Pilsen, Charles University in Prague and Faculty Hospital in Pilsen, alej Svobody 80, Pilsen, 304 60 Czech Republic; Faculty Hospital in Pilsen, Central Immunoanalytical Laboratory, alej Svobody 80, Pilsen, 304 60 Czech Republic

**Keywords:** Predictive, Preventive and personalized medicine, Statistics, Training, Educational tools, EPMA, Recommendations

## Abstract

The main goal of personalized medicine is the individualized approach to the patient’s treatment. It could be achieved only by the integration of the complexity of novel findings in diverse “omics” disciplines, new methods of medical imaging, as well as implementation of reliable biomarkers into the medical care. The implementation of personalized medicine into clinical practice is dependent on the adaptation of pre-graduate and post-graduate medical education to these principles. The situation in the education of personalized medicine in the Czech Republic is analyzed together with novel educational tools that are currently established in our country. The EPMA representatives in the Czech Republic in cooperation with the working group of professionals at the Faculty of Medicine in Pilsen, Charles University in Prague have implemented the survey of personalized medicine awareness among students of Faculty of Medicine in Pilsen—the “Personalized Medicine Questionnaire”. The results showed lacking knowledge of personalized medicine principles and students’ will of education in this domain. Therefore, several educational activities addressed particularly to medical students and young physicians were realized at our facility with very positive evaluation. These educational activities (conferences, workshops, seminars, e-learning and special courses in personalized medicine (PM)) will be a part of pre-graduate and post-graduate medical education, will be extended to other medical faculties in our country. The “Summer School of Personalized Medicine in Plzen 2015” will be organized at the Faculty of Medicine and Faculty Hospital in Pilsen as the first event on this topic in the Czech Republic.

## Review

Personalized medicine (PM) is the novel model of individual patient’s medical care [1, 2]. The main goal of PM is the shift from the concept of “one medicine fits to all patients with the same disease” to individual treatment of each patient—“the right treatment to the right patient in the right time” [3–6].

Personalized medicine is based on the evolving knowledge about the human genome, gene functions and the genetic basis of the individual differences in responses to a treatment. However, without a “societal stimulus”, the evolution of the personalized medicine would not be most probably occur. The basis of this “societal stimulus” was the alarming finding, in the 90th years of the last century, that the disease incidence and mortality induced by the treatment intolerance and complications (adverse drug reaction) are superior to the disease incidence and mortality caused by civilization diseases [6]. Consequently, a boom occurred in the use of genomics, metabolomics, proteomics and other omics methods for the prediction of the adverse drug reactions.

The strategy of PM is to provide an individualized approach to each patient, based on his/her personal genetic profile and combining information from omics disciplines with innovative preventive and therapeutic strategies that are more efficient, safe and cost-effective [7–9]. The philosophy of PM has become a reality with the sequencing of human genome and the development of novel technologies including laboratory diagnostics, advances in genetics and genomics, new methods of medical imaging and implementation of various biomarkers into the medical care [10–13]. The process of personalization of the health care includes an efficient prevention and screening, more complex and targeted diagnostics, prediction of possible adverse health effects of prescribed drugs, individualization of therapy and treatment monitoring. This can be achieved by means of prediction, prevention or diagnostics biomarkers, genetic testing of individuals, advances in pharmacogenomics and so on.

The personalized medicine has already proved its usefulness in clinical practice and will be the most important trend in the future medicine. The potential of genetics and genomics to provide new horizons for prevention, diagnosis and treatment of disease is immense, but in order to use this appropriately, and to prevent misuse, before the vision of a personalized medicine can be fully realized, health professionals as well as medical trainees must be given the proper educational foundation [14, 15].

### The role of education in personalized medicine

Traditional medical education needs to be modified in order to prepare medical fellows and health professionals to the challenges of personalized medicine implementation. Several issues to be considered are listed:

1. As personal genetic information will become a current component of a patient’s record, it is crucial that medical students be trained to use and interpret this information appropriately and responsibly [16]. *Fundamental training in genetics and genomics*, along with the attendant legal, ethical and psychosocial issues, should fall within the purview of medical school education.
2. *Pharmacogenetics* and *pharmacogenomics* should be incorporated in medical curricula. There is a growing need to prepare clinicians and health providers to the anticipated arrival of pharmacogenomics diagnostics tools (companion diagnostics) [17, 18].
3. Diseases are complex; they originate from a combination of genetic and environmental factors. Also, patients are complex entities resulting from the integration of environmental elements, genetic characteristics, and individual mutations. Recent omics technologies produce a large amount of data. A complex approach is needed, with the use of databases and models (bioinformatics), and students should be trained to work *in a multidisciplinary team* [19].

Over the world, a great number of universities already offer undergraduate and graduate education in molecular medicine, in some of which personalized medicine is also discussed. However, the majority of medical schools have not yet incorporated genetic or genomic courses into their curricula. Yet, there are a few exceptions, such as programs dedicated to personalized medicine at Duke University (NC, USA) and at Mount Sinai School of Medicine (NY, USA) [20]. Also, some schools have updated their curricula by including genomic medicine, such as the Harvard Medical School (MA, USA). Some of these courses are designed for e-learning, for example, the US National Coalition for Health Professional Education in Genetics has developed a series of web-based medical education programs discussing the influence of genetics on various diseases [21]. Similarly, in the UK, the National Genetics Education and Development Centre has developed evidence-based learning objectives and competencies in genetics for health professionals [20].

There is also a debate about the most efficient educational models that could be deployed. At Stanford School of Medicine, a novel hands-on genomics course was developed in 2010 that provided students the option to undergo personal genome testing as part of the course curriculum [22]. Authors had hypothesized that the use of personal genome testing in the classroom would enhance the learning experience of students. After the course, authors concluded that undergoing personal genome testing and using personal genotype data in the classroom enhanced students’ self-reported and assessed knowledge of genomics.

### Education in PM in the Czech Republic

The medical pre-graduate and post-graduate educational system in the Czech Republic has very long history. It was oriented mainly toward the traditional medicine. During the past decade, the system changed due to the step by step reveal of new findings in biosciences (mentioned above). However, a complex approach to the PM education focused on its principles does not exist yet. Therefore, the implementation of PM into the common medical education as well as into the clinical practice is essential. The education is thought to be most effective among young physicians and medical students.

In the Czech Republic, there are eight faculties of medicine attached to four universities, faculties of health sciences (for nurses, health care, and laboratory staff) and one faculty of pharmacy. The faculties of medicine (and also faculties of natural sciences) have already incorporated issues linked with personalized medicine in their curricula. The courses dealing with personalized medicine, molecular medicine, systems biology or pharmacogenomics are facultative and offer specialized lectures; however, their quality differs among universities/faculties, they are scarcely interconnected with the overall curriculum, and practical training is missing.

In the next paragraphs, the road to an implementation of the PM education into the curriculum at the Faculty of Medicine in Pilsen is presented.

### Survey of PM awareness among students of the Faculty of Medicine in Pilsen

**Figure 1:**
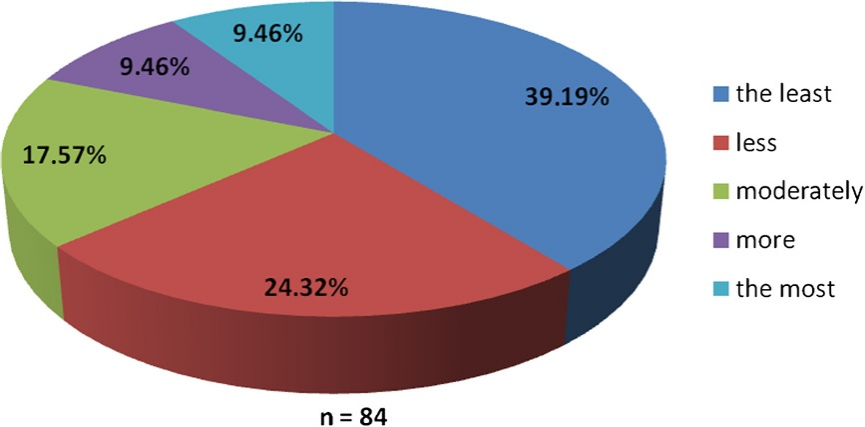
**The PM questionnaire results 1.** The PM questionnaire results—the question “Have you ever heard the term personalized medicine?”

**Figure 2:**
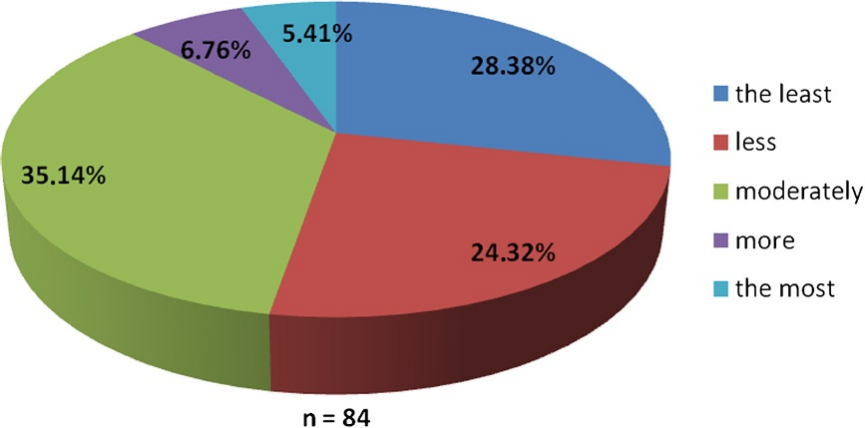
**The PM questionnaire results 2.** The PM questionnaire results—the question “Would you be able to explain, what does the personalized medicine mean?”

**Figure 3:**
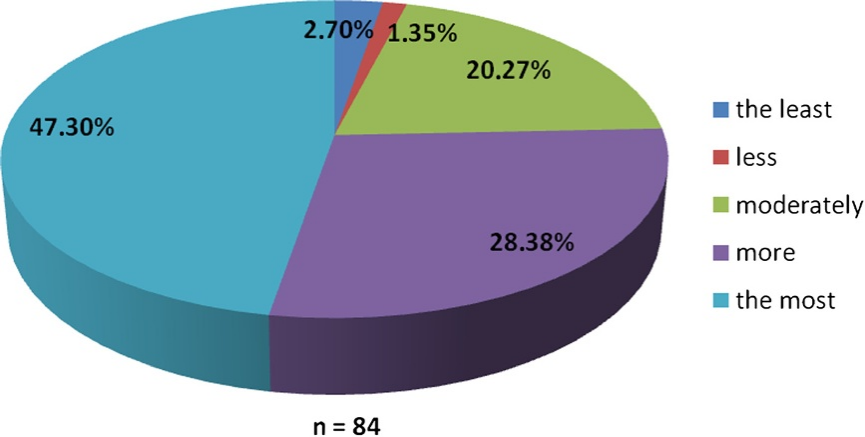
**The PM questionnaire results 3.** The PM questionnaire results—the question “Do you consider PM to be important?”

**Figure 4:**
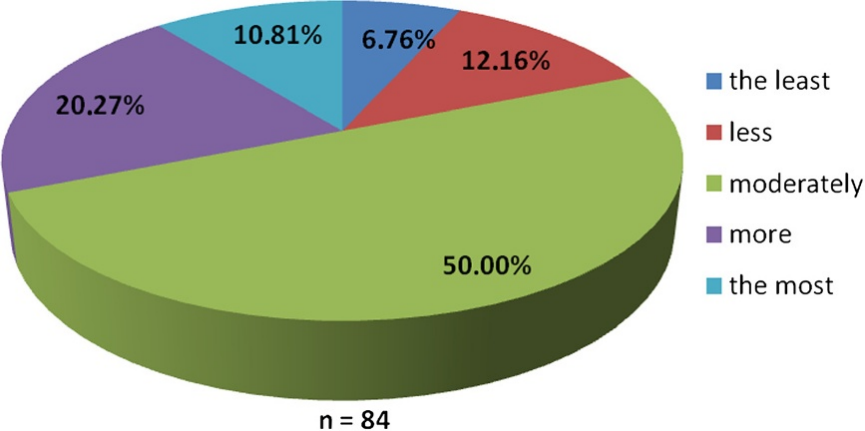
**The PM questionnaire results 4.** The PM questionnaire results—the question “Do you think personalized medicine should be studied as an independent discipline or through the various disciplines separately?”

**Figure 5:**
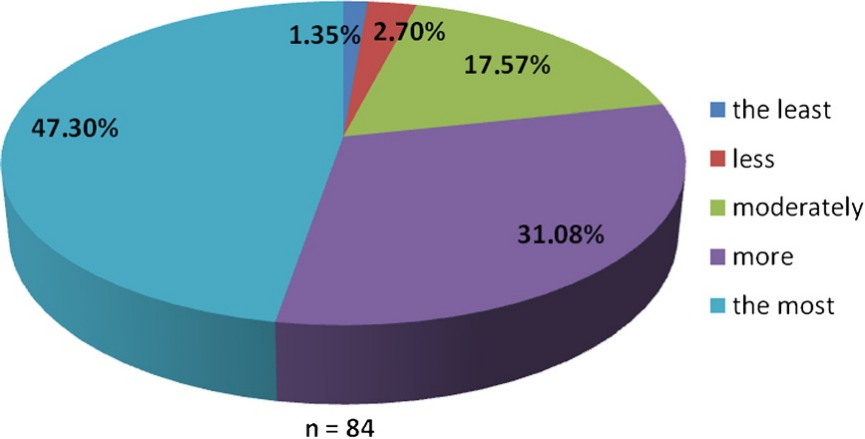
**The PM questionnaire results 5.** The PM questionnaire results—the question “Do you consider the role of personalized medicine will increase with the progress of knowledge?”

**Figure 6:**
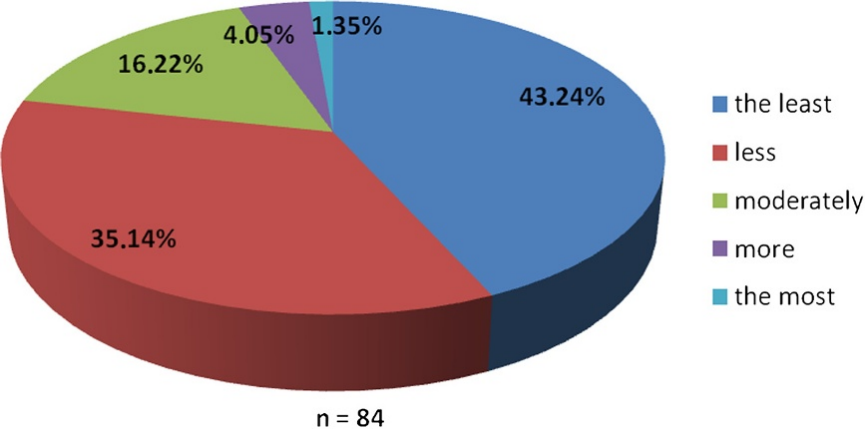
**The PM questionnaire results 6.** The PM questionnaire results—the question “Is tuition in personalized medicine sufficient in pre-graduate education in medical school?”

**Figure 7:**
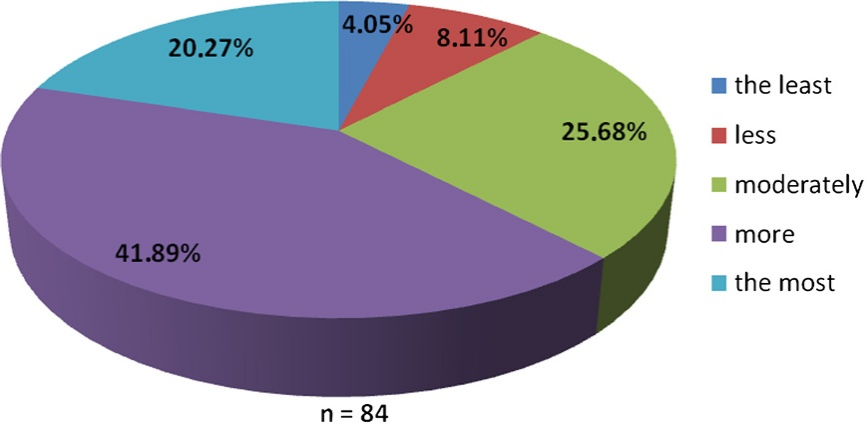
**The PM questionnaire results 7.** The PM questionnaire results—the question “Do you think personalized medicine should be implemented into the pre-graduate education of medicine for all students?”

**Figure 8:**
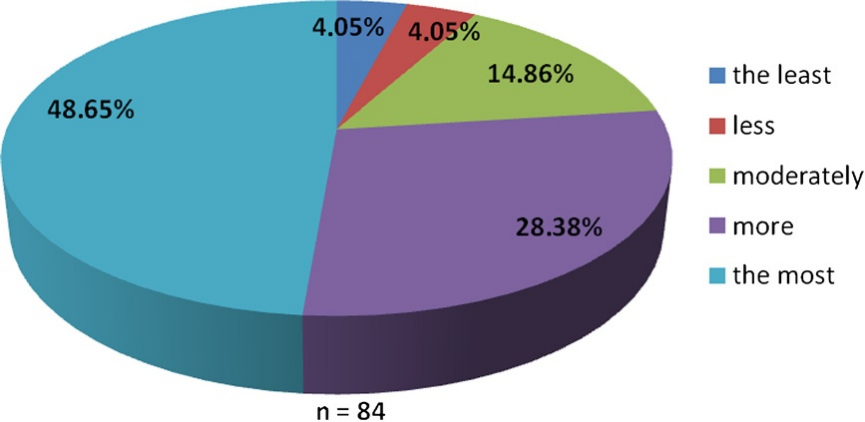
**The PM questionnaire results 8.** The PM questionnaire results—the question “Do you think personalized medicine should be implemented into the pre-graduate education of medicine as credit course?”

**Figure 9:**
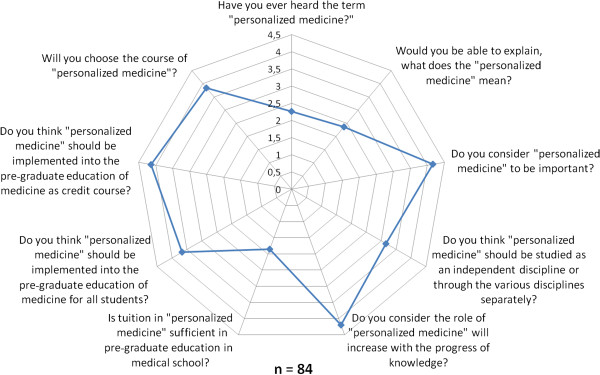
**The PM questionnaire results 9.** The PM questionnaire results—means of responses for particular questions.

The working group of professionals at the Faculty of Medicine in Pilsen, Charles University in Prague and in the Faculty Hospital in Pilsen has been established with the goal to implement the principles of PM into the pre-graduate and post-graduate education at these institutions. The first step was to ascertain the real situation—how intense was the knowledge of PM among medical students. For this reason, the “Personalized Medicine Questionnaire” was prepared and addressed to medical students at the Faculty of Medicine in Pilsen. Students had to answer eight nominal questions about the PM. Each question had the response range from 1 to 5 (from “the least” up to “the most”). There were 84 responders, mainly students from the fourth year of medical school. The distributions of responses for each question are represented on the Figures 1, 2, 3, 4, 5, 6, 7 and 8. The summary of the means of responses for each question is represented in Figure 9 and Table 1. The results showed lacking knowledge of PM principles and students’ will of education in PM.

**Table 1:** **The summary of the means of responses for each question in the personalized medicine questionnaire**

Question in personalized medicine questionnaire	The mean of responses (1–5)
Have you ever heard the term personalized medicine?	2.26
Would you be able to explain, what does the personalized medicine mean?	2.36
Do you consider personalized medicine to be important?	4.16
Do you think personalized medicine should be studied as an independent discipline or through the various disciplines separately?	3.16
Do you consider the role of personalized medicine will increase with the progress of knowledge?	4.2
Is tuition in personalized medicine sufficient in pre-graduate education in medical school?	1.85
Do you think personalized medicine should be implemented into the pre-graduate education of medicine for all students?	3.66
Do you think personalized medicine should be implemented into the pre-graduate education of medicine as credit course?	4.14

Each question had the response range from 1 to 5 (from “the least” up to “the most”).

As the results of the PM questionnaire show, the awareness about PM among students is quite weak (more than 39% have not even heard the term “personalized medicine”), although most students (more than 75%) recognize the importance of PM and would welcome its implementation into the pre-graduate education.

### Educational activities in PM at the Faculty of Medicine in Pilsen

Several educational activities (workshops, conferences, seminars, new optional courses) addressed particularly to medical students and young physicians at our institution were realized in last 5 years.

Since 2009, the presentations concerning PM and its applications have been included in the scientific program of the Immunoanalytical Days—the congress with an international participation, organized every year by the Czech Society of Nuclear Medicine. Since 2011, PM topics have their independent section at this congress. In 2011, a two-day conference “Personalized medicine—from bench to bed” was held at the Faculty of Medicine in Pilsen. The topics of presentations were varying from strategic ones (Horizon 2020 presented by Dr. Patrick Kollar from the European Commission, and Innovative Medicine Initiative) through PM overviews from the USA experts, to the examples of research topics and case studies from cardiology, oncology, neurology and so on.

In 2013, a conference “Genes determine treatment” was organized with the cooperation of the EPMA. Several members of EPMA including the President of EPMA Dr. V. Costigliola and EPMA Secretary-General Prof. Dr. O. Golubnitschaja participated at this event and students from the Faculty of Medicine have a first-hand opportunity to learn about the activities of the association and its members. The presentations embraced many issues, including bioinformatics, systems biology, company diagnostics and so on. The presentation of special topics on the Czech personalized medicine website was the next step in the support of education. This website is widely accessible and free of charge.

### Perspectives

One of the main activities of the working group of professionals in the personalized medicine domain at the Faculty of Medicine in Pilsen and in Faculty Hospital in Pilsen is the organization of the first course of the “Summer School of Personalized Medicine in Pilsen 2015”, the first event in the Czech Republic on this topic. During two weeks of the courses, the theoretical background and clinical application of PM across the variety of the fields of medicine will be presented and discussed. The summer school will be open for the pre-graduate as well as post-graduate students of medicine from the Charles University and from other medical schools in the Czech Republic and for medical students abroad.

### Recommendations

PM has become an increasingly important topic for physicians, health-care organizations, and their patients. The knowledge and education in PM is crucial for the efficiency of future medical care. The best and the most effective way to achieve this goal is to educate the students of medicine together with young health-care professionals in this field. The PM education should be an essential part of medical study, should be comprehensive and complex, respect mutual interrelations, and should be based on the most advanced knowledge of molecular genetic cause of diseases and their sophisticated scientific management. Various forms of educational activities are optimal, such as conferences, workshops, e-learning and special courses in PM.

## Conclusions

The concept of personalized medicine is one of the most perspective trends in medicine at present. PM assures the individual approach to each patient with tailored therapy and distinctive medical care. The education in this field is the keystone for understanding and application of PM principles. The educational activities of the working group of professionals in the PM at the Faculty of Medicine in Pilsen, Charles University in Prague and in the Faculty Hospital in Pilsen are unique through the country and will be extended among pre-graduate and post-graduate medical students in the Czech Republic.
